# Bone Marrow Aspirate in the Treatment of Chondral Injuries

**DOI:** 10.3389/fsurg.2016.00033

**Published:** 2016-06-16

**Authors:** James Holton, Mohamed A. Imam, Martin Snow

**Affiliations:** ^1^Queen Elizabeth Hospital, University Hospitals Birmingham, Birmingham, UK; ^2^Royal Orthopaedic Hospital, Birmingham, UK; ^3^Suez Canal University, Ismailia, Egypt

**Keywords:** bone, marrow, aspirate, concentrate, cartilage

## Abstract

The ability of mesenchymal stem cells (MSCs) to transdifferentiate into a desired cell lineage has captured the imagination of scientists and clinicians alike. The limited ability for chondrocytes to regenerate in chondral injuries has raised the concept of using MSCs to help regenerate and repair damaged tissue. The expansion of cells in a laboratory setting to be delivered back to the patient is too costly for clinical use in the present tough economic climate. This process is slow with due to the complexity of trying to imitate the natural environment and biological stimulation of chondral cell replication and proliferation. Bone marrow aspirate concentrate (BMAC) has the potential to provide an easily accessible and readily available source of MSCs with key growth factors that can be used in treating chondral injuries. This review summarizes the underlying basic science of MSCs and the therapeutic potential of BMAC.

## Introduction

The development of techniques to harvest and amplify specific cell lines using mesenchymal stem cells (MSCs) has generated huge interest in trauma and orthopedic surgery due to its potential for tissue repair and regeneration (1).

Historically, chondral defects in the young patient have been treated with microfracture and mosaicplasty. The resulting fibrocartilage production, rich in type I collagen is structurally inferior to the hyaline cartilage in joints but may delay the onset of osteoarthritis (2). We have state-of-the-art arthroplasty options to those patients who develop end-stage osteoarthritis. However, there is a clear demand for regenerative techniques to slow or even reverse this disease process.

Stem cells have been of interest to the reconstructive surgeon, as they have the potential for differentiation into the cell lineage of interest (3). Embryonic stem cells are perhaps the holy grail of stem cells, as they are totipotent and have the potential to differentiate into any cell lineage (3). However, there are considerable ethical issues and considerations, as they require harvest from embryonic tissue. Adult stem cells show some level of differentiation and develop into cells that support the tissue of origin, which is the logical sequence as these stem cells can help repair and regenerate tissues in their vicinity (3). These cells can be stimulated to irreversibly change their cell lineage in a process termed transdifferentiation, where cells transform into a different cell type (3, 4). Bone marrow aspirate (BMA) with its relative ease of harvest provides a less controversial source of MSCs with the required properties for use in regenerative orthopedics. Our aim is to review this literature regarding the basic science of bone marrow-derived stem cells and the therapeutic potential of bone marrow aspirate concentrate (BMAC) in the regeneration and repair of chondral injuries.

## Articular Cartilage

Articular (hyaline) cartilage is designed to allow low-friction articulation and to resist repetitive loading of the joint. It consists of extracellular matrix with water, collagen, and proteoglycans surrounded by chondrocytes (5). It has a unique structure with differing orientation of fibers that alter the physical properties. The superficial horizontal fibers help to resist shear forces, while the deeper vertically orientated fibers resist compressive forces through the joint (5, 6).

Hyaline cartilage has very little regenerative capacity in part to the avascular nature of the hyaline cartilage and that it relies on diffusion from synovial fluid for nutrition (7). In partial-thickness injuries, the avascular nature of the cartilage prevents the repair and healing process (7). Thus, even small cartilage lesions can remain, accelerate wear due to increased point loading and propagate. In full-thickness injuries, there is stimulation of blood flow from the underlying subchondral bone allowing the formation of fibrocartilage at the injury site (7, 8). The fibrocartilage may slow degeneration but as the biomechanical properties are inferior to hyaline cartilage it may still lead to established osteoarthritis and joint degeneration (9).

## Traditional Treatment Strategies

Treatment strategies are frequently age dependent. As we age the population of MSCs decreases and hence reduces the repair and healing potential of the elderly (10). This combined with the already limited regenerative capacity of chondrocytes means that elderly patients are most frequently treated conservatively with the aim of relieving symptoms with the use of analgesics and physiotherapy-guided exercise programs. This can buy time until the requirement of joint replacement surgery. In the younger active population, cartilage damage is primarily treated surgically. Techniques, such as microfracture, aim to stimulate local blood flow by penetrating subchondral bone to allow MSCs to exit and access the focal chondral defect to aid healing (11). Mosaicplasty is the autologous harvest of an osteochondral graft from a non-weight bearing area and transfer to the primary defect (12). This ultimately produces a mixture of hyaline and fibrocartilage (13). The fibrocartilage is biomechanically inferior to the native hyaline cartilage (2). Hence, there is a demand for a more physiological repair process to enable patients to avoid the degenerative sequelae associated with chondral damage.

Autologous chondrocyte implantation (ACI) was initially thought to be very promising, using chondrocytes from non-weight bearing areas of the joint, expanding them in culture, and delivering them back to the area of damage. However, it comes at the financial cost of cell expansion and requiring a two-stage procedure alongside the donor site morbidity and the production of hyaline-like cartilage (14). This process has not shown clear benefit when compared with either microfracture or mosaicplasty in the short term, with Lim et al. finding no difference at 1 year (15). A Cochrane review in 2010 by Vasiliadis and Wasiak reviewed six small randomized control trials that compared ACI with either mosaicplasty or microfracture (16). The study found no conclusive evidence ACI was superior to mosaicplasty or microfracture (16). A 2012 systematic review of 14 articles by Rodriguez-Merchan also found no difference between ACI, microfracture, and mosaicplasty (17). However, contrary to the earlier reviews, Bentley et al. have shown that ACI was superior to mosaicplasty with reduced failure rates and better outcomes long term (>10 years) (18). This work has been supported by Biant et al. in 2014 reporting favorable results of ACI for large chronic chondral and osteochondral defects of the knee with 10-year follow-up (19). Biological augmentation with MSCs may help improve long-term outcomes.

## Mesenchymal Stem Cells

Mesenchymal stem cells are found not only in bone marrow but also in many other mesenchymal tissues, including adipose, bone, synovium, and blood (20). However, although MSCs from these different mesenchymal tissues have a similar phenotype, they differ in their differentiation potential and this is likely to reflect their host tissue (21). They are formed from colony-forming units, which are phenotypically fibroblast like (CFU-F) (22, 23).

The MSCs’ ability to transdifferentiate into the desired cell lineage is fundamental to the regenerative process, but these cells also have very important local paracrine affects to alter their local microenvironment to conditions favorable for regeneration and repair (24). Cell-to-cell communication is integral to the normal wound-healing response and allows recruitment and migration of MSCs into the required area due to upregulation of specific cell-surface receptors (25). Once in the vicinity, the exact interplay of MSCs to the local environment is not fully understood, but it is clear that the MSCs are able to modulate all stages of the normal wound-healing response (26). This is due to downregulation of the inflammatory cytokines, including interleukin 1 (IL-1), interleukin 6 (IL-6), interferon-γ, and tumor necrosis factor alpha (TNF-α) (26, 27). Aggarwal and Pittenger found an increase in anti-inflammatory cytokines interleukin-10 (IL-10) and interleukin-4 (IL-4) when human MSCs were cultured with immune cells (28). As the bone marrow-derived MSCs are innate, they have the appropriate host major histocompatibility complex (MHC) allowing them to avoid destruction by the immune system (27).

## Isolation and Expansion of MSCs

Isolation of MSCs has traditionally been done by utilizing a unique feature of these cells in that they are adherent to plastic surfaces (29). Although this seems useful, unfortunately, it does allow some cellular contamination with cells that do not have the same propensity to differentiate and undergo clonal expansion (30). The International Society for Cellular Therapy states that MSCs must express the surface markers CD105, CD73, and CD90 and lack CD45, CD34, CD14, CDIIb, CD79α, or CD19 (20).

In order to identify and select MSCs that can undergo clonal expansion and differentiate, surface markers have been used. Many of the recognized markers for MSCs from the International Society for Cellular Therapy have been targeted using monoclonal antibodies. There is now established monoclonal antibodies to CD73 (31), CD105 (32), CD90, and STRO-1 (33). The development of monoclonal antibodies specific to MSC markers have allowed separation of MSCs from other hematopoetic cells. However, the markers are not specific enough to different subpopulations of MSCs, and indeed, there is no single specific marker (29, 30, 33). As no technique has been completely successful, it represents the heterogeneity of bone marrow-derived MSCs and shows the complexity of the signaling and regulation of these cells for differentiation (33, 34).

These techniques have allowed crude identification of bone marrow-derived MSCs. There is great clinical interest in understanding the molecular biology of MSCs, so that it can be manipulated for clinical use. Of great importance is the ability of MSCs to differentiate and proliferate *in vitro*. This has great potential as cells from the individual could be expanded and reimplanted to avoid immunological activation (23).

Mesenchymal stem cell differentiation is regulated by interplay with a number of growth factors and pathways, including the fibroblast growth factors (FGFs), insulin-like growth factors (IGFs), and the wingless-type (WNT) signaling pathway (35). However, the most dominant factor is the transforming growth factor-beta (TGF-β). These TGF-β ligands bind to their specific serine/threonine kinase-linked cell-surface receptors. This causes a downstream cascade of SMAD protein phosphorylation, generating a heterocomplex of phosphorylated SMAD proteins. This complex can enter the cell nucleus, promoting the generation of transcription factors involved in chondrocyte differentiation (35).

The key transcription factor for chondrogenesis is the sex-determining region Y-box 9 (SOX-9) protein (36). This is the end result of the mitogen-activated protein kinase (MAPK) pathway, which is activated by the extracellular signal-related kinases (ERKs), the C-june-NH2-terminal kinases (JNKs), and the P38 MAPK pathway (35). Downstream signaling results in the phosphorylation of SOX-9, and its transfer to the nucleus to promote chondrogenesis.

The proliferation of MSCs is primarily under the control of the canonical WNT/β catenin signaling pathway (37). The signaling pathway involves WNT-protein binding to frizzled (Fz) receptors initially, which leads to the inhibition of glycogen synthase kinase-3β (GSK-3β) allowing β-catenin translocation to the nucleus to induce gene expression and subsequent cellular proliferation (38). *In vitro* expansion of MSCs does have some inherent drawback, including loss of stem characteristics and chondrogenic differentiation potential (39). However, there is now evidence that manipulation with FGF-2 and inhibition of WNT signaling during differentiation can help increase proliferation rates and promote chondrogenesis, respectively (39, 40).

There has been interest in manipulating specific growth factors or signaling molecules in these pathways to help develop bone marrow-derived MSCs *in vitro*. Gene therapy has been used to manipulate favorable conditions for chondrocyte differentiation (41). The growth factor IGF-1 has proven particularly useful to promote differentiation and chondrogenesis but has a short-lived effect due to rapid and effective clearance (2). This has been overcome by Frisch et al. using recombinant viral vectors to human bone marrow cell *in vitro* to provide sustained IGF-1 locally and facilitate differentiation and chondrogenesis (2). This process has also been used with TGF-β and SOX-9 using recombinant adeno-associated virus (rAAV) vectors (42). *In vitro* culture of MSCs with bone morphogenetic proteins (BMPs), FGF-1, and IGF-1 have all enhanced chondrogenesis in a laboratory setting (43). However, none of this has transferred to the clinical arena.

The clonal expansion and proliferation of cells *in vitro* comes at a financial cost that may render it unfeasible in the tough economic climate and the endless cost cutting of today’s health-care system. BMA has been studied as a low-cost source of MSCs that may augment the repair and regeneration of musculoskeletal tissue.

## Isolation and Preparation of BMA

To overcome the considerable financial cost of *in vitro* cell expansion, unprocessed BMA has been used as a source of bone marrow-derived MSCs (44). There are a number of potential areas to harvest BMA. Hyer et al. compared the iliac crest, tibia, and calcaneus and assessed the number of MSCs (45). The iliac crest provided a higher mean concentration of MSCs when compared with the other sites. However, with increasing age, there is reduction in absolute number of MSCs with a reduced proliferation capacity, which may have implications in treating the elderly population (46, 47).

Batinic et al. reviewed the number of MSCs from the first 1 ml and subsequent samples from the iliac crest (48). In subsequent samples, the nucleated cell population and CFU level were 3 and 10× lower than the first 1 ml of aspirate (48). Muschler et al. showed that as the volume of aspirate from the iliac crest increases from 2 to 4 ml, the number of MSCs decreases by 50% (49). A recent study by Peters and Watts in horses has shown that needle advancement of 5 mm up to three times can increase the proportion of MSCs, although subsequent passes did not provide additional benefit (50). This is likely due to hemodilution with aspirated blood.

Approximately 0.001% of nucleated cells from BMA are MSCs (51). In an attempt to increase the proportion of MSCs, the aspirate is concentrated to produce BMAC. This is most commonly performed by centrifuging the aspirate (22). Hernigou et al. has shown a direct correlation between increased concentration of MSCs and increased rates of healing in 60 patients with established non-unions of the tibia (52).

## Biological Cell Scaffolds

There have been many studies looking to augment the delivery of BMAC and MSCs to the area of concern. Biological scaffolds have been explored to primarily fill a defect and provide a stable microenvironment and framework in which new tissue can develop (53). These scaffolds can be biologically engineered to enhance the microenvironment, such as carrying specific growth factors to promote chondrogenesis (54).

Scaffolds can be natural or synthetic in various forms either solid in fibers, sheets, mesh, or powder or a semi-solid gel, hydrogel, or glue form (53, 55). The most frequent naturally occurring materials are hyaluronic acid, collagen, agarose, alginate, and fibrin, whereas polylactides are the most commonly used synthetic material (53, 55). These scaffolds can be delivered through mini-open procedures, although most are performed arthroscopically.

Kon et al. reviewed 305 scaffold-based procedures up to 2013 with 127 studies in clinical trials (56). There were a huge variation in different combinations of cells with scaffolds and scaffolds alone. This highlights there is no clear consensus of what the optimum method is. It is clear that the scaffold needs to be cost-effective, reproducible, and provide an environment that allows cellular differentiation and integration with the host.

## Preclinical and Clinical Outcomes of BMAC in Chondral Injuries

There are a number of established animals’ models using expanded bone marrow-derived MSCs and a biological scaffold as a vector to augment chondrogenesis with good results (57, 58). This work has been extended in studying BMAC in the animal model.

Fortier et al. reviewed 12 horses with 1.5 cm^2^ full-thickness cartilage defects treated with microfracture and microfracture plus BMAC (59). At 8 months, there was improved defect filling, type II collagen production and integration in the BMAC group compared with microfracture alone (59). McIlwraith et al. found similar results in 10 horses with 1 cm^2^ defects in 2 knees treated with microfracture (60). At 1 month, one of the knees was injected with BMAC. At 12 months, the knee injected with BMAC showed better macroscopic repair and raised concentration of aggrecan (the main proteoglycan in articular cartilage) (60).

Current clinical studies have shown that BMAC have been useful for the treatment of small lesions with various scaffolds to augment delivery. Enea et al. studied patients undergoing microfracture covered with a resorbable composite of natural hyaluronan matrix and synthetic polyglycolic acid with BMAC (61). At 12 months, the lesions were macroscopically normal with hyaline-like tissue production and MRI confirming defect filling (61).

Gobbi et al. treated 15 patients with grade IV cartilage lesions with BMAC on a collagen matrix with 2-year follow-up (62). There were improvements in the International Knee Documentation Committee (IKDC), Visual Analog Scale, Knee injury and Osteoarthritis Outcome score (KOOS), Lyshold, Marx, SF-36, and Tegner scores. Biopsy of these patients revealed hyaline-like tissue at repeat arthroscopy at 2 years (62). Gobbi et al. also studied the effect of BMAC with a hyaluronan-based scaffold in patients less than and greater than 45 years of age (63). Functional outcome scores were not significantly different between the two groups, highlighting the potential in treating defects in older patients. Histological analysis yielded hyaline-like tissue (63). Giannini et al. used BMAC on a hyaluronic acid membrane to treat osteochondral defects of the talus (64). This was compared with open and arthroscopic ACI. All three groups improved in American Orthopedic Foot and Ankle Society (AOFAS) score with no statistical difference between groups and evidence of hyaline-like tissue. BMAC had the advantage of being an arthroscopic, single-step procedure (64).

Biological grafts have also been trialed to augment delivery of BMAC. Krych et al. used BMAC on a demineralized bone graft to treat cartilage defects of the knee compared with platelet-rich plasma (PRP) on the same scaffold (65). The BMAC group had improved cartilage maturation and filling of the defects as shown on MRI (65). Centeno et al. has studied BMAC with and without an adipose tissue grafts in knee arthritis (66). Both groups had improved numerical pain scores and lower extremity functional scale scores, but unfortunately, there was no apparent benefit from the adipose graft itself (66).

Skowroński et al. (67) has demonstrated beneficial effects of BMAC on large (4–12 cm^2^) chondral lesions in the knee in 54 patients. At 1 year, there was an average improvement of 25 points on the KOOS and 35 points on the Lysholm score.

The success of BMAC in large chondral defects has led to investigation into BMAC in the treatment of osteoarthritis. Kim et al. has shown that the size of the lesion and age of the patients are important considerations for treatment with BMAC (68). The study found a smaller chondral defects (up to 6 cm) and patients <60 years of age had better outcomes when administrating BMAC for knee osteoarthritis (68). This is in keeping with MSC decline with age (69). Although defect size and age are important, Kim et al. has shown that simple joint injection with BMAC improved functional quality of life scores in elderly patients (mean 60.7 years and range 53–80 years) with established knee osteoarthritis (70). The scores included the IKDC, KOOS, SF-36, and the Lysholm score that were all improved at 3, 6, and 12 months postoperatively. Thus, highlighting the potential of BMAC in slowing the time required for arthroplasty.

## Conclusion

The long-term outcomes of traditional treatment modalities versus treatment with bone marrow-derived MSCs in the form of BMAC need to be reviewed alongside the relative cost-effectiveness of each. However, it is clear that BMAC has great potential in the repair and regeneration of chondral damage. There is clear need for further investigations to establish the best way to isolate, prepare, and deliver BMAC to the chondral injury. This should allow standardization in the use of BMAC to provide the best possible outcomes for patients.

## Author Contributions

JH is the main author for the manuscript. He has designed, drafted, and revised the manuscript along with its submission. After the initial draft was submitted, MI has worked to revise and edit the latest manuscript. Prof. MS has provided senior review, edited, and revised the manuscript.

## Conflict of Interest Statement

The authors declare that the research was conducted in the absence of any commercial or financial relationships that could be construed as a potential conflict of interest.
